# Older adults and healthcare professionals have limited awareness of the link between the Mediterranean diet and the gut microbiome for healthy aging

**DOI:** 10.3389/fnut.2023.1104238

**Published:** 2023-01-27

**Authors:** Lauren O’Mahony, Emma O’Shea, Eibhlís M. O’Connor, Audrey Tierney, Mary Harkin, Janas Harrington, Sharon Kennelly, Elke Arendt, Paul W. O’Toole, Suzanne Timmons

**Affiliations:** ^1^Centre for Gerontology and Rehabilitation, School of Medicine, University College Cork, Cork, Ireland; ^2^Department of Biological Sciences, University of Limerick, Limerick, Ireland; ^3^Health Research Institute, University of Limerick, Limerick, Ireland; ^4^APC Microbiome Ireland, Alimentary Pharmabiotic Centre, University College Cork, Cork, Ireland; ^5^School of Allied Health, University of Limerick, Limerick, Ireland; ^6^Health Implementation Science and Technology Research Cluster, Health Research Institute, University of Limerick, Limerick, Ireland; ^7^Age and Opportunity, Dublin, Ireland; ^8^School of Public Health, University College Cork, Cork, Ireland; ^9^National Primary Care Strategy and Planning, Health Service Executive, Dublin, Ireland; ^10^School of Food and Nutritional Sciences, University College Cork, Cork, Ireland; ^11^School of Microbiology, University College Cork, Cork, Ireland

**Keywords:** gut microbiota, Mediterranean diet, healthcare professionals, older adults, healthy aging, aging, microbiome, science communication

## Abstract

**Objectives:**

Strategies to improve the gut microbiome through consuming an improved diet, including adopting the Mediterranean Diet (MD), may promote healthy aging. We explored older adults’ and healthcare professionals’ (HCPs) perspectives of the MD, gut health, and microbiome for their role in healthy aging.

**Design:**

Phenomenological qualitative.

**Setting:**

Community-dwelling older adults and HCPs in primary and secondary care in Ireland.

**Participants:**

Older adults (aged 55 + years), recruited through social, retirement and disease-support groups. HCPs recruited through researcher networks and professional associations.

**Measurements:**

Semi-structured 1:1 interviews and focus groups (FGs) conducted remotely with older adults and HCPs separately. Interviews/FGs were recorded, transcribed, and coded using inductive thematic analysis.

**Results:**

Forty-seven older adults were recruited (50% male; 49% aged 60–69 years; 28% 70 +), and 26 HCPs including dietitians (*n* = 8); geriatricians (*n* = 6); clinical therapists (*n* = 4); nurses, pharmacists, catering managers, and meal-delivery service coordinators (*n* = 2 each). Older adults considered the MD “*a nice way to enjoy food*,” good for cardiovascular health and longevity, but with accessibility and acceptability challenges (increased salads/fish, different food environments, socio-cultural differences). HCPs felt the MD is included in healthy eating advice, but not overtly, mostly through the promotion of mixed-fiber intake. Older adults considered “live” yogurt and probiotics, and to a lesser extent fiber, to maintain a “healthy gut,” suggesting the gut has “*something to do with”* cognitive and digestive health. Overall, microbiota-health effects were considered *“not common knowledge*” among most older adults, but becoming more topical among both professionals and the public with advancing scientific communication.

**Conclusion:**

While “gut health” was considered important, specific effects of the MD on gut microbiota, and the significance of this for healthy aging, was under-recognized. Future efforts should explain the importance to older adults of maintaining the gut microbiota through diet, while appreciating perspectives of probiotic products and supplements.

## 1. Introduction

Nutrition and diet play a key role in promoting health and contributing to a healthy aging process (1, 2). In particular, the Mediterranean Diet (MD) is suggested as a dietary strategy for addressing multiple age-related issues such as frailty and neurodegenerative decline (3, 4). The MD involves a varied wholefood diet, with higher consumption of fiber through fruits, vegetables and legumes, as well as unsaturated fats through nuts, olive oil and fish, and less red meat and dairy (5). Adopting the MD may facilitate healthy aging by promoting a favorable gut microbiota profile, modulating the gut-brain axis and preventing age-related decline (2, 6, 7).

Recently, the NU-AGE dietary trial specifically showed that the MD contributed to optimal gut microbiota, better global cognitive ability and episodic memory, reduced bone loss, and improved immune function and blood pressure among a cohort of older adults from five countries (8). Diet alters gut microbiota in a way which impacts upon the rate of health decline in older age (9, 10), mediating the gut-microbiota and immune system interplay (11). Consequently, the potential translation of these findings to health advice is advancing to actionable recommendations. For example, a personalized diet has been proposed for people with neurodegenerative disease to positively influence microbiota signatures (12). Furthermore, with changes in gut microbiota potentially influencing serotoninergic function in older age (13), the therapeutic potential for improving mental health *via* gut microbiota modulation has also gathered much attention (14, 15).

Despite potential for improved health through optimized gut microbiota, there are barriers to incorporating the MD in Northern Europe, including the perceived difficulty of adopting the diet while living in a colder climate, cultural differences, demand for convenience, or overall acceptability (16–18). There are differences in the foods typically consumed as part of the MD and Irish diet, as has been described previously (17). For instance, the current Irish diet is relatively low in fruit, vegetables, fish and nuts/seeds, with olive oil used in very small amounts, but relatively high intake of red meat, poultry, dairy, processed grains, and confectionary (19). Barriers to promoting maintenance of gut microbiota may also exist. One study of consumer receptivity to clinical microbiota interventions in a Middle-Eastern population found that the role of the gut microbiota in health, particularly the gut–brain axis, was poorly understood in both scientific and public communities (20). Experts have previously noted the need for clinical intervention studies which prove the role of microbiota as a key aspect in health in order for adapted medical and public health education (21).

To our knowledge, no study has been carried out in an Irish population of current awareness and perspectives of the MD and gut microbiota in the context of healthy aging. We thus explored older adults (55 + years of age) and healthcare professionals’ (HCPs) perspectives of the MD and gut microbiota for their role in healthy aging, using qualitative methods. This enquiry forms part of a wider project which aims to develop a novel food product by harnessing key nutrients of the MD to promote optimal gut microbiota in older age. Exploring stakeholder perceptions through qualitative enquiry can frame the context for implementing targeted dietary intervention through functional food products, as well as wider promotion of healthy eating strategies within a Western European cohort.

## 2. Materials and methods

This study followed a phenomenological qualitative design and is reported in adherence to COREQ guidelines (22) (Supplementary material). A phenomenological approach was considered appropriate by the researchers to yield an in-depth exploration of older adults’ and HCPs’ lived experience and perception of the MD and gut microbiota in relation to healthy aging (23). Consideration was also given to different qualitative health research methodologies used in nutrition research, with the principles of qualitative description closely aligned to the chosen research methods (24). Full ethical approval has been granted (SREC 2021-079).

### 2.1. Recruitment and data collection

Adults aged 55 + years (“55 + adults”) were recruited through social, community and retirement groups, and disease-specific support groups. The researcher (LoM; female research assistant trained in qualitative methods) contacted organizations, requesting to share study information with members as appropriate (email, newsletters, social-media, word-of-mouth). Interested participants were invited to contact the researcher. Purposive sampling included a gender balance and range of ages, and inclusion of people with relevant diseases (stroke, arthritis, etc.). HCPs/stakeholders were recruited through researcher networks and relevant national professional associations. There were no sampling criteria for HCPs/stakeholders. Recruitment continued until data saturation was reached.

Interviews and focus-groups were conducted with 55 + adults and HCPs in Ireland from July 2021 to January 2022. Two semi-structured schedules were developed by the research team, for 55 + adults (Supplementary File 1) and for HCPs/stakeholders (Supplementary File 2). Three pilot interviews were conducted with 55 + adults and the schedule was refined accordingly. LoM attended a Public Patient Involvement (PPI) meeting with an age advocacy and activity organization (Age and Opportunity) to explore perspectives during recruitment and data collection (e.g., saying “food” rather than “diet” to avoid confusion with a restrictive or prescribed diet). The schedule for HCPs/stakeholders was used flexibly to suit professional roles. Participants chose a one-off 1:1 interview or focus-group to accommodate personal preferences and time commitments. Written consent, informed by a participant information leaflet, was obtained. Interviews/focus-groups were conducted remotely using Microsoft Teams^®^ and recorded using the software’s inbuilt function, or by telephone and audio-recorded using the research laptop. Recordings were assigned numerical codes and erased following transcription. Anonymized transcripts were used for analysis. LoM conducted all interviews/focus-groups and transcribed 80% of data, with assistance from the research team. LoM had no prior relationship with study participants, who understood her as the study’s research assistant. Field notes were taken; data not transcribed by LoM were read through before coding to ensure data familiarization.

### 2.2. Data analysis

Inductive thematic analysis was used (25), supported by NVivo 12. LoM coded an initial subset (15%; 6/41) of transcripts using a data-driven approach before applying these codes to remaining transcripts, with additions/revisions as required. Four transcripts were reviewed by a second coder (EoS; female senior researcher), to ensure quality and consistency. Throughout the coding process, a list of themes and sub-themes was derived, adapted, and refined. For demographic data, valid percentages are reported.

## 3. Results

A total of 47 adults aged 55 + and 26 HCPs took part. This included 25 interviews (17 adults aged 55 +, and 8 HCPs), along with 16 focus-groups, each with 2–5 participants (30 adults and 18 HCPs took part this way). Interviews/focus-groups lasted, on average, 45?min with 55 + adults and 43 min with HCPs. Six focus-groups with 55 + adults proceeded with only two participants due to the other planned participants’ unexpected non-attendance.

Participant demographics are outlined in Table 1. Most 55 + adults were aged 60–69 years (*n* = 23; 48.9%), with four aged 80 + years, and equal numbers of males (*n* = 23) and females (*n* = 23). One person identified as non-binary. Most were recruited *via* social/community or retirement groups (*n* = 33), and several through disease support groups e.g., stroke, Parkinson’s disease, and arthritis (*n* = 11). Three were recruited from an organization within the Traveling community, a particularly hard-to-reach ethnic group (26). There were more female (*n* = 21; 81%) HCPs than males (*n* = 5; 19%), with occupations described below.

**TABLE 1:** Demographic characteristics of study participants (*N* = 73).

		*N* (%)
*Participant type*	Older adult (55 + adults)	47 (64.4)
	Healthcare professional	26 (35.6)
*Age (55 + adults)*	<60	11 (23.4)
	60–69	23 (48.9)
	70–79	9 (19.1)
	80 +	4 (8.6)
*Employment status* *(55* + *adults)*	Retired	28 (59.6)
	In employment or self employed	19 (40.4)
*Residential province* *(All participants)*	Connacht	5 (7.0)
	Leinster	35 (49.3)
	Munster	30 (42.3)
	Ulster	1 (1.4)
*HCP occupation*	Dietitian	8 (30.7)
	Geriatrician	6 (23.1)
	Clinical therapist*	4 (15.4)
	Pharmacist	2 (7.7)
	Clinical nurse (one manager; one nurse specialist)	2 (7.7)
	Catering manager (one community-based; one residential)	2 (7.7)
	Meal delivery service coordinator	2 (7.7)

*Speech and language therapist (*n* = 2), physiotherapist (*n* = 1), occupational therapist (*n* = 1).

**FIGURE 1:**
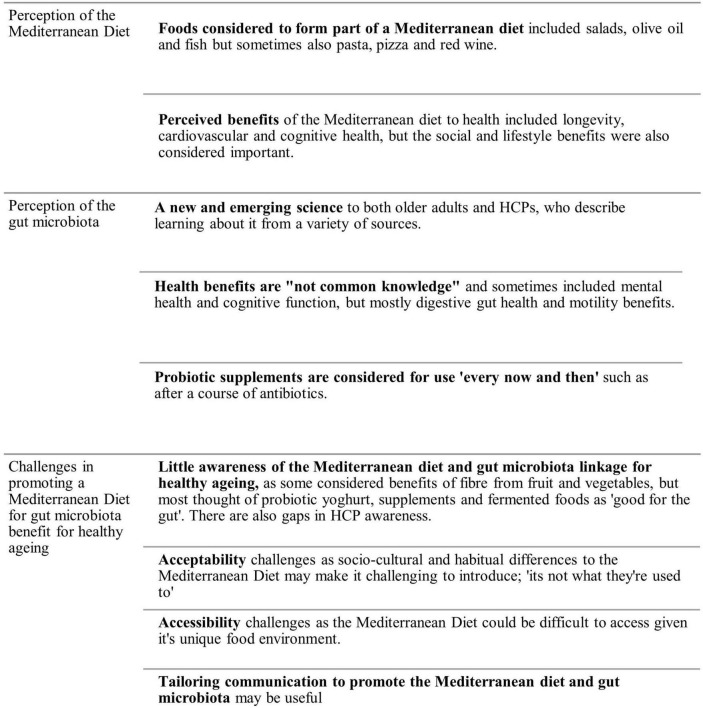
Themes identified in the data organized by domain.

Nine key themes were identified in the data which could be organized into three domains, an overview of which is described below (Figure 1). Participant quotations are presented to illustrate thematic findings, attributed to the 55 + adults (abbreviated as OA) and HCPs (disciplines included). An overview of quotations can be found in Supplementary File 3.

### 3.1. Perception of the Mediterranean diet

#### 3.1.1. Foods considered to form part of the MD

Most 55 + adults understood foods included in the MD, though there were some areas of uncertainty. HCPs did not specifically discuss older adults’ understanding of the MD composition but suggested it may depend on whether the person had traveled to Mediterranean countries, and that the MD “*is not what they’re (older person) used to”* (see subtheme “Acceptability: Socio-cultural and habitual differences of the MD”). Indeed, many 55 + adults associated the MD with foods eaten in Mediterranean countries, including fresh salads, olive oil and fish, and noted the visual attraction of colorful and seasonal fruit and vegetables, speaking favorably of home-growing and local production. However, some felt that the MD was “*vague*,” and were uncertain that all perceived components could be regarded as beneficial to health. Some considered pasta, pizza, breads and wine may also be consumed in the MD; *“I wouldn’t think it includes all the red wine and sugars*… *even pastas*… *a clear definition of the MD is hard to get*… *Is it just vegetables?*… *and lots of oil?” (OA#30).*

While some were aware there might be “*limited amounts of meat*” and more fish or plant-based dishes, few discussed reduced red meat or dairy as a feature of the MD. Some were concerned about achieving sufficient protein intake if not eating meat regularly, or believed meat was needed for sustenance. Some commented on the inclusion of nuts, beans, legumes, and wholegrains while others did not; “*I suppose they eat a lot of fish, and they grow their own food. I don’t know necessarily what the food is, I think there’s nuts involved?” (OA#4)*.

Several 55 + adults used olive oil and spoke of cod-liver oil supplements for Omega-3; “*our mother used to spoon us cod liver oil*… *(it’s) good for your bones and builds you up” (OA#55).* Others had “*heard something about*” taking a spoonful of olive oil to “*stave off dementia*” or that oil is carcinogenic if burnt. Several understood the difference between saturated and unsaturated fats within the MD, while others were divided on the healthfulness of fats; *“I always think oil is unhealthy, even olive oil*… *that’s just oil? you’re eating lots of fats?” (OA#4).* One person considered that all fats were part of the MD “…*in the MD, it’s ok to eat cheese, use cream, butter” (OA#16).*

#### 3.1.2. Perceived benefits of the Mediterranean diet to health

The MD was considered by both HCPs and 55 + adults to have cardiovascular and longevity benefits, with few people noting benefits for cognitive health; *“I think there’s some place in Italy where they all live to a hundred*… *it’s supposed to be something to do with their diet” (OA#20).* Another had doubts about the evidence; *“from a consumer’s point of view, I never saw any evidence that it (MD) will extend my life” – (OA#30).* Many 55 + adults felt the MD could help to prevent heart disease and was good for lipid profiles; *“I think that it has beneficial effects on, let’s say, if you have high cholesterol” (OA#28)*. One person, living with arthritis, suggested the diet could reduce inflammation.

While HCPs were not explicitly asked about the health benefits of the MD (as it was assumed most would be familiar with this), most described it generally in terms of healthy eating, while only some cited its role for specific diseases; *“there’s most information there with regards to prevention of dementia” (Dietitian #4)*. Some dietitians and geriatricians spoke about what they felt was “*relatively weak*” evidence from epidemiological study of the MD and disease outcomes in older cohorts; *“The problem with diet and relating it to chronic illness prevention, the studies are far few and between and not robust in some cases” (Dietitian #4).* Others had questions about the preventative potential of MD foods alone in achieving health benefits in an older population; *“I would have thought the evidence starts with these populations where it is their whole food diet that they’ve had that’s helped to prevent against whatever diseases” (Dietitian #5).* For people already living with moderate to severe neurodegenerative disease, geriatricians were most concerned about “*whatever [nutrients] you can get into them (patients)*,” particularly adequate energy and protein intake, rather than a MD approach.

Both 55 + adults and HCPs suggested that the MD could have social and wellbeing benefits. One geriatrician suggested that the MD may have benefits due to its social structure; “… *certain lifestyles or behaviours that are not related specifically to the type of food that you eat*… *but can be around how you organise mealtimes” (Geriatrician #6).* Separately, another questioned whether the diet was still beneficial *“if you extract the social component” (Pharmacist #15).*

### 3.2. Perceptions of the gut microbiota

#### 3.2.1. The gut microbiome is a new and emerging science

Some 55 + adults spoke about their increased awareness of the microbiota in recent years; *“I know that there’s a lot of research going on now on the gut microbiome*… *and I would wonder about certain things, would they be helpful for me*… *like the probiotics” (OA#10).* They have heard about the gut microbiota from a variety of sources including product advertisement, TV or radio, health professionals, scientists, books, or their own research; *“I’ve heard a lot about it on the radio*… *dietitians and nutritionists and people talking about how important it is to have a balanced microbiome” (OA#5).* Several others were aware of the microbiome from research in a nearby university: “*in (University College Cork)*… *doing terrific work on the gut*… *relating it to the brain and depression*” *(OA#28).*

HCPs acknowledged the abundance of new discoveries around the microbiota, with one dietitian noting they have been “*struggling to keep on top of it” (Dietitian #3).* Another felt research advancements were important because of the role of the gut microbiome in mental health; *“You see a lot of people with irritable bowel syndrome*… *related to anxiety and the gut*… *I think having that research about the gut microbiome is really important for all round health, not just for digestive health, but mental health” (Dietitian #3).*

#### 3.2.2. Perceived health benefits are “not common knowledge”

Both HCPs and 55 + adults felt effects of the gut microbiota on different aspects of health were “*not common knowledge”* among most older adults. There appeared only a vague understanding of the gut-brain association among 55 + adults; *“I don’t quite understand the relationship between healthy gut and healthy body, and not ageing and dementia*… *how far is it actual proved scientific knowledge*… *that the healthy gut does prevent dementia?” (OA#63).* A few discussed how different foods affect their mood, such as confectionary and convenience foods. Others considered the gut microbiota had “something to do with” cognitive health*; “I have read about it*… *there’s some bacteria in the brain*… *but I let the details go” (OA#64).* For others, the association with dementia was “*a new angle*,” noting they *“wouldn’t have made the connection with dementia.” (OA#52)*.

More frequently, 55 + adults related the gut microbiota or “healthy gut” to digestive health and motility, for instance “*an absence of indigestion and digestive disorders*,” or the “*mechanical”* breakdown of food. Dietitians also considered bowel health and the effects of fiber; “*you would be looking at fibre and fluid intake, not necessarily very probiotic effective food, but just the overall fibre their diet provides” (Dietitian #7).* One geriatrician discussed the potential interest in probiotic foods like yogurts for their preventive potential against antibiotic-associated diarrhoea. Many participants recognized yogurts to have gut benefits; *“Anything I see in the line of live cultures*… *yoghurts and things*… *I don’t know what it actually means but I presume it’s something fairly good” (OA#46)*. Several 55 + adults thought of a “*gut cleansing*” or “*clearing”* effect, suggesting fasting was part of maintaining gut health “*you go 12 hours to 14 hours with no food, isn’t that another way of doing that*… *clearing your gut?” (OA#42).* A couple discussed eating linseeds to “*help take out some of the*… *poisons*” from the gut.

It was suggested that maintaining the gut microbiota is seen as a local issue rather than something with systemic effects; “*there’s so many things that it affects*… *but they’re (older adults) not aware of the other general health benefits to everything, that it’s not just gut*” *(Dietitian #1).* Only some considered wider effects, for instance, one person with arthritis had some understanding of the microbiome’s role in inflammation; “*there’s some good bacteria and bad bacteria and if the good bacteria win, you won’t have leaky gut and you won’t have inflammation” (OA#2).* One dietitian was interested in immunity benefits from maintaining gut microbiota, particularly in the context of COVID-19, while another considered the microbiota as important when looking at “*overall health*” of an older cohort and “*not just their malnutrition risk and management*” *(Dietitian #4)*.

#### 3.2.3. Probiotic supplements are considered for use “every now and then”

Many 55 + adults associated a healthy microbiota with taking probiotic supplements; *“I look after my biome by*… *taking a supplement and eating live yoghurt” (OA#21)*. They suggested their sporadic and restorative use; “*the antibiotics have done so much damage to your system and then the probiotics, you take them to counteract that” (OA#1).* Some 55 + adults implied that gut health was only something to be concerned about every now and then; *“my gut is probably a bit leaky, as we get older*… *so every so often I’ll take a product that is meant to kind of help repair the gut a bit” (OA#25)*. Several indicated they would be more aware of gut health if they suffered from issues like constipation or irritable bowel syndrome, suggesting they don’t worry about gut health because they don’t have any trouble with it; *“Am I going to start changing the way I eat to try and tweak my gut microflora?*… *I don’t think I’ve got a problem, so I don’t think I need to do anything about it” (OA#3)*.

One geriatrician described that they were “*cynical*” of probiotic products; “… *anecdotally, the probiotics are dead by the time they reach your small intestine*… *where they’re needed.” (Geriatrician #5).* One dietitian felt instead that fruit and vegetables were best “*avenue*” for benefiting the gut microbiota over supplements which they felt can be “*quite an extreme measure.” (Dietitian #2).*

### 3.3. Challenges in promoting a Mediterranean diet for gut microbiota benefit for healthy aging

#### 3.3.1. Little awareness of the Mediterranean diet and microbiota linkage for healthy aging

Few participants, including HCPs, specifically discussed the role of the MD in optimizing gut microbiota profiles. Those who did discuss any association between the two mostly suggested digestive gut health and motility could be optimized through the consumption of dietary fiber, mainly by intake of fruits, vegetables, and salads. For instance, several 55 + adults spoke about how a “*balanced and varied*” diet of colorful fruit and vegetables could be beneficial for gut health. Others felt fruit, nuts and seeds were good to “*keep the tummy active*” or that “*fibre is what feeds the microbiome.*” While participants recognized the microbiota benefit from yogurts, one person didn’t feel yogurts fit into the MD; “… *I don’t think I’d associate yoghurts now with Mediterranean diet but apparently, they’re supposed to be good for your gut” (OA#20)*.

Some 55 + adults felt there were microbiota benefits to be gained from diets other than the MD, such as the Japanese or Korean diet due to its fermented food content. Many 55 + adults described that they ate foods such as kombucha, kimchi, sauerkraut, and kefir *“because of the good bacteria*” *(OA#64).* These diets were considered by one person as healthier than the MD; “*The ultimate healthy diet, I think you’d be looking at Japanese macrobiotic sort of diet*… *Whereas the Mediterranean diet*… *it’s a nice way to enjoy food” (OA#19)*

#### 3.3.2. Acceptability: Socio-cultural and habitual differences of the Mediterranean diet; “it’s not what they’re used to”

Some 55 + adults and HCPs felt that implementing the MD in Ireland might be difficult to align with existing cultures and traditions; “*it’s something different from what we’d be used to*” *(physiotherapist #10).* The Mediterranean style of eating late in the evening might not suit Irish traditions, where for many older adults the main meal is typically eaten mid-day; *“I’m not sure that many Irish people would be able to cope with eating as late in the evening as they do” (OA#24)*. Several others felt Irish cultures around high meat and dairy consumption may make it hard to introduce some of the plant-based or fish elements of the MD; *“I’m not a great fan of oily fish*… *I’m more a meat and two veg sort of fellow” (OA#21).* Others commented on the Irish Catholic tradition of having fish on Friday, and that the idea of having fish any other day “*might need a little bit of encouragement” (Meal-delivery service coordinator #17).*

There were several perceived differences between Mediterranean salads vs. in Ireland where many salads have rich sauces or are starch-based; “… *potato salad and coleslaw. I think in Ireland a lot of people see that as a salad” (OA#1).* Some 55 + adults felt that adopting salads as a dietary staple would be difficult because of the colder climate; “*no cold food*… *your gut doesn’t like it in the winter” (OA#23).* There was a sense that what we have in Ireland can be ‘just as good’ as the MD; *“what’s wrong with having an Irish diet and looking at our own seasonal foods and maybe bringing something that doesn’t have to travel as far” (OA#26).*

#### 3.3.3. Accessibility: The Mediterranean diet in different food environments

Both 55 + adults and HCPs felt the MD might be difficult to access in Ireland in terms of produce availability and location of retail outlets relative to where older people lived. Produce sold in Mediterranean open-air food markets were considered local, fresh, affordable and of better quality than imported produce; “*The fruit and veg, seasonality*…. *if you’re in the Mediterranean, there would be more opportunities to eat fresh, it’s more accessible” (Dietitian#4).* One person felt that more support for horticulture in Ireland is needed to make salads more accessible, while someone else described fresh fish as less accessible if living inland. Others, including HCPs, felt access to the MD might be impeded by cooking requirements, cost, and acquisition of multiple ingredients; *“The affordability of a MD and the effort required to accumulate the parts of it might be a barrier for people on reduced income and with people with reduced mobility. Maybe not everything is readily available in their local corner store” (Pharmacist #15).*

#### 3.3.4. Tailoring communication to promote the Mediterranean diet and gut microbiota

Some 55 + adults and HCPs discussed how language could help to implement the MD. Sometimes, the word “diet” was seen as requiring an entire dietary restriction; *“if I went on the Mediterranean diet*…*” (OA#3); “*… *does it have to be full Mediterranean diet or can it be incorporated?” (OA#S20)*. Several dietitians noted how their advice on the MD would be “*subtle*”; *“It’s overlapping with*… *increase fish, get your fruit and veg in there. It’s indirectly Mediterranean without calling it that” (Dietitian #1).* Others spoke about how they use “*small steps*” to explain components of the MD instead of “*diving into olive oil, chickpeas, and lentils*…*” (Dietitian #3).* Similarly, one dietitian noted how they would tend to explain the *“simplified version*” of the gut microbiome; “… *it’s not necessarily something I mention to them, but it’s in my head*… *if they were talking about the good stuff in the yoghurt*… *I would talk about the probiotic, again not the technical terms” (Dietitian #1).*

## 4. Discussion

This study explored Irish older adults’ and HCPs’ perspectives of the MD, gut health, and microbiome in terms of supporting healthy aging. Both stakeholder groups considered the importance to health of the MD and gut microbiota separately, with little recognition of the specific role of the MD in promoting a favorable gut microbiota profile and thus contributing toward healthy aging. There were varying perceptions of the link between gut microbiota and gut health, how to maintain it, and which physiological and aging processes it may affect. Many older adults in particular perceived the gut microbiota role mainly in terms of digestive health rather than overall physical or cognitive health. Older adults and professionals perceived challenges in promoting the MD to an older population in Ireland.

Several 55 + adults and HCPs perceived there to be a beneficial role of fiber from fruits, vegetables, salads, nuts and seeds in promoting gut health. However, many associated gut microbiota maintenance with products such as “live” yogurts or probiotic supplements and did not specifically reference the MD or Mediterranean foods. This perception that probiotic supplements and products are required to maintain gut health suggests the influence of marketing in shaping public perceptions. Some 55 + adults perceived there to be microbiota benefits from other diets like the Asian diet, for a similar contribution to longevity as the MD, *via* its fermented food components. While the literature can support both of these perspectives (27, 28), there may be a missed opportunity for the promotion of the role of the MD in gut microbiome health and its involvement with healthy aging processes (8). In addition, some 55 + adults were of the opinion that a fasting effect of gut “*cleansing”* or *“clearing”* was beneficial for gut health. Despite some research in this area (29), these perspectives highlight the need for differentiation of “gut microbiome” and “gut health” in the public perception and experience (30).

Participants recounted learning about the gut microbiota from various sources such as health and nutrition professionals and consumer-directed advertising. There appeared to be a blurred distinction between digestive “gut health” and “microbiota health,” as well as self-perceived knowledge gaps. For instance, one dietician suggested they were “*struggling to keep on top of*” gut microbiota research, pointing to the potential for additional professional education activities to increase HCP knowledge. Many 55 + adults and HCPs discussed bowel motility or digestive conditions when asked about the gut microbiota, with only a handful suggesting that there could be systemic benefits to immunity or inflammation, with little regard for age-specific effects. Thus, it appeared that gut health is often perceived as a local issue and not something which affects the wider body. The perspectives observed in our sample are not uncommon. A cross-sectional survey distributed among adults in the United Arab Emirates found that while respondents understood a basic definition of the microbiota, they lacked understanding of its role in disease protection and immunity, with HCP knowledge not significantly higher than that of non-HCPs (31).

Linked to this, gut health was seen to be only in need of attention “*every now and then*,” perhaps after a course of antibiotics or if someone was concerned about digestion. This may partly reflect the increased promotion of and demand for probiotics and prebiotics from consumers (32), and indeed their perceived sporadic usage. One study showed that despite recommendations for probiotic foods and supplements to be used consistently (33), up to 30% of people who use probiotics do so only intermittently or in an *ad hoc* manner (34, 35).

As research increasingly demonstrates the importance of diet, and particularly the MD (8), for gut microbiota benefit (36), public and professional receptivity to the MD as a dietary strategy for healthy aging is important. Many 55 + adults considered the MD as healthy and recognized cardiovascular and longevity benefits, consistent with research messaging (5). Many described their familiarity and experience with various food components of the diet, such as salads, olive oil, and fish. However, our study suggests adaptability challenges for the MD in Ireland. For instance, the perception among some 55 + adults that foods like pasta and pizza equate to the MD diet, and their varying perspectives on olive oil or fish oil as either having benefits for brain health (“staving off dementia”) or as being “*unhealthy*” or “*carcinogenic*,” suggests a poor understanding of the MD and its key constituents. There were also recurring suggestions that the MD is just “*a nice way to enjoy food*,” with benefits only attributing to a “*whole-diet approach*” rather than its components.

While some dietitians pointed out that the MD is already *“subtly”* included in healthy eating advice, consideration of evidence-based health benefits from the MD for older adults as demonstrated in published observational and trial data were not to the fore. Some dietitians and geriatricians suggested that epidemiological study of the effects of Mediterranean foods in older cohorts had “*not covered itself in glory.*” Indeed, the longevity effects of the MD were considered difficult to “unbundle” from the effects of lifestyle practices (37). This is despite adherence to Mediterranean dietary patterns being associated with longevity, reduced risk of overall mortality, cardiovascular diseases, overall cancer incidence, and neurodegenerative diseases (2).

Many participants appeared to consider the MD as an “all-or-nothing” approach; the concept of adopting some elements of the MD as a solution to acceptability and accessibility challenges did not arise. While there are many unique socio-cultural elements to the Mediterranean way of eating (38), both older adults and HCPs in this study considered experiential factors such as dietary meal staples and the timing of the meals as potentially challenging to align within existing long-standing traditions. Participants also felt accessibility issues might inhibit the diet in a non-Mediterranean region, considering unique food environments or the need to overcome availability barriers in different consumer retail and supply-chains (16). Such differences have indeed been noted in the literature (16, 39). Overall, the apparent “all-or-nothing” perception of implementing the MD, combined with existing HCP opinions of epidemiological evidence, may be limiting the perceived promotion and acceptability of MD adoption in a non-Mediterranean culture and environment.

To promote adoption of the MD in an older cohort, efforts might focus on increasing stakeholders’ knowledge of what constitutes a MD and what does not, as well as public and professional understandings of the health benefits associated with the MD. For instance, it has been already suggested to incorporate the key principles of the MD into current Irish Food Pyramid guidance (17). Health promotion should include information on the gut microbiota in terms of its effects on body health, and not just “gut health.” Public health promotion campaigns, as well as targeted education for health and nutrition professionals, may be useful in this regard.

### 4.1. Strengths and limitations

We involved an adequate sample size, appropriate gender balance and range of ages. There was specific inclusion of 55 + adults from a range of social, retirement and disease-specific groups, including people from potentially marginalized groups such as the Traveling community and LGTBQ + community (26). While we collected information such as age, gender, area of residence and employment status (retired/not) from the older adults, we did not collect racial-ethnic data, education level or socioeconomic status, which is a limitation.

Most of the HCPs recruited were predominantly in contact with older adults with established disease, and we acknowledge the absence of primary care HCPs such as GPs and public health nurses (PHNs) who may have more experience with fitter, community-dwelling older adults. Thus, there may have been a mismatch between our older person sample and older people as envisioned by the HCPs. Professional networks known to the research team were contacted, but due to an ongoing protracted COVID-19 wave, no GPs or PHNs were available to participate. Nevertheless, exploring the views of 55 + adults and HCPs simultaneously allowed identification of important potential knowledge gaps for future health promotion and science communication.

We acknowledge the limitations of a qualitative study in terms of data generalizability, and we recommend that the themes determined in this study are explored further in a large population survey.

## 5. Conclusion

Older adults and HCPs describe being increasingly aware of research examining how the gut microbiota relates to aging processes and the interplay which exists with diet; however, the translation of research findings to public and professional understanding appears incomplete. This is not entirely unexpected. There appears to be knowledge gaps in both stakeholder groups in recognizing that the Mediterranean diet promotes gut microbiota profiles linked to healthy aging. While individuals considered the effects of fiber through fruits and vegetables and probiotic yogurts and supplements in promoting digestive health, there was little awareness of the systemic effects of the MD *via* promoting beneficial microbiota in terms of preventing disease in old age. Some references were made to inflammation, mental health and immunity, however, disparities remained toward the role of the MD in promoting healthy gut microbiota signatures. Separately, challenges in perception, acceptability, and accessibility may exist in implementing the MD in a non-Mediterranean culture and environment. These insights should be considered in future science communication and health promotion efforts to improve public and professional understanding of the role of the MD in promoting the gut microbiota for healthy aging.

## Data availability statement

The datasets presented in this article are not readily available because participants were only asked for their consent to quotation/publication of extracts from the focus-groups and interviews. They did not give their consent to having any other data made publicly available or shared with other research groups. Requests to access the datasets should be directed to corresponding author.

## Ethics statement

This study and all procedures involving research participants were approved by the Social Research Ethics Committee at University College Cork (SREC/2021/079). Written informed consent was obtained from all study subjects prior to their participation in interviews/focus groups via a participant information leaflet which included details of the study, the right to withdrawal, data protection policies and procedures, and a consent form attached. Verbal consent was confirmed and recorded at the start of each interview/focus group.

## Author contributions

ST, EA, and PO’T conceived and designed the study. LO’M collected the data. LO’M checked and coded the transcripts with assistance from EO’S. LO’M conducted and interpreted the analyses with guidance from EO’S and ST. LO’M drafted the manuscript with input from EO’S and ST. EO’C, AT, MH, JH, and SK acted as external expert advisors on the project. All authors reviewed, provided feedback on, and approved the final draft of the manuscript.

## References

[1] Mak T Louro Sandra C. *The role of nutrition in active and healthy ageing for prevention and treatment of age-related diseases: evidence so far.* Luxembourg: Publications Office of the European Union (2014).

[2] Katz D Meller S. Can we say what diet is best for health? *Annu Rev Public Health.* (2014) 35:83–103. 10.1146/annurev-publhealth-032013-182351 24641555

[3] Sugimoto T Sakurai T Ono R Kimura A Saji N Niida S Epidemiological and clinical significance of cognitive frailty: A mini review. *Ageing Res Rev.* (2018) 44:1–7. 10.1016/j.arr.2018.03.002 29544875

[4] Singh B Parsaik A Mielke M Erwin P Knopman D Petersen R Association of mediterranean diet with mild cognitive impairment and Alzheimer’s disease: a systematic review and meta-analysis. *J Alzheimers Dis.* (2014) 39:271–82. 10.3233/JAD-130830 24164735 PMC3946820

[5] Trichopoulou A Martínez-González M Tong T Forouhi N Khandelwal S Prabhakaran D Definitions and potential health benefits of the mediterranean diet: views from expers around the world. *BMC Med.* (2014) 12:112. 25055810 10.1186/1741-7015-12-112 PMC4222885

[6] McGrattan A McGuinness B McKinley M Kee F Passmore P Woodside JV Diet and inflammation in cognitive ageing and Alzheimer’s disease. *Curr Nutr Rep.* (2019) 8:53–65. 10.1007/s13668-019-0271-4 30949921 PMC6486891

[7] Ghosh T Shanahan F O’Toole P. The gut microbiome as a modulator of healthy ageing. *Nat Rev Gastroenterol Hepatol.* (2022) 19:565–84. 35468952 10.1038/s41575-022-00605-x PMC9035980

[8] Ghosh T Rampelli S Jeffery I Santoro A Neto M Capri M Mediterranean diet intervention alters the gut microbiome in older people reducing frailty and improving health status: The NU-AGE 1-year dietary intervention across five European countries. *Gut.* (2020) 69:1218–28. 10.1136/gutjnl-2019-319654 32066625 PMC7306987

[9] Claesson M Jeffery I Conde S Power S O’connor E Cusack S Gut microbiota composition correlates with diet and health in the elderly. *Nature.* (2012) 488:178–84. 22797518 10.1038/nature11319

[10] An R Wilms E Masclee A Smidt H Zoetendal E Jonkers D. Age-dependent changes in GI physiology and microbiota: Time to reconsider? *Gut.* (2018) 67:2213–22. 10.1136/gutjnl-2017-315542 30194220

[11] García-Montero C Fraile-martínez O Gómez-Lahoz A Pekarek L Castellanos A Noguerales-Fraguas F Nutritional components in western diet versus mediterranean diet at the gut microbiota-immune system interplay. implications for health and disease. *Nutrients.* (2021) 13:699. 10.3390/nu13020699 33671569 PMC7927055

[12] Milošević M Arsić A Cvetkoviæ Z Vučić V. Memorable food: fighting age-related neurodegeneration by precision nutrition. *Front Nutr.* (2021) 8:688086. 10.3389/fnut.2021.688086 34422879 PMC8374314

[13] O’Mahony S Clarke G Borre Y Dinan T Cryan J. Serotonin, tryptophan metabolism and the brain-gut-microbiome axis. *Behav Brain Res.* (2015) 277:32–48. 10.1016/j.bbr.2014.07.027 25078296

[14] Butler M Mörkl S Sandhu KV Cryan J Dinan T. The gut microbiome and mental health: what should we tell our patients?: le microbiote intestinal et la santé mentale: que devrions-nous dire à nos patients? *Can J Psychiatry.* (2019) 64:747–60. 10.1177/0706743719874168 31530002 PMC6882070

[15] Verma H Phian S Lakra P Kaur J Subudhi S Lal R Human gut microbiota and mental health: advancements and challenges in microbe-based therapeutic interventions. *Indian J Microbiol.* (2020) 60:405–19. 10.1007/s12088-020-00898-z 33087991 PMC7539250

[16] Moore S McEvoy C Prior L Lawton J Patterson C Kee F Barriers to adopting a mediterranean diet in Northern European adults at high risk of developing cardiovascular disease. *J Hum Nutr Diet.* (2018) 31:451–62. 29159932 10.1111/jhn.12523

[17] Tierney A Zabetakis I. Commentary changing the irish dietary guidelines to incorporate the principles of the mediterranean diet?: proposing the medéire diet. *Public Health Nutr.* (2018) 22:375–81. 10.1017/S136898001800246X 30319088 PMC10260643

[18] Haigh L Bremner S Houghton D Henderson E Avery L Hardy T Barriers and facilitators to mediterranean diet adoption by patients with nonalcoholic fatty liver disease in Northern Europe. *Clin Gastroenterol Hepatol.* (2019) 17:1364–71.e3. 10.1016/j.cgh.2018.10.044 30391437

[19] Williams M Lannetta P Styles D. *A combined environmental and nutri-economic assessment of diets.* (2020). Available online at: www.true-project.eu (accessed December 21, 2022).

[20] Bendriss G Al-Ali D Shafiq A Laswi I Mhaimeed N Salameh M Targeting the gut microbiome: A brief report on the awareness, practice, and readiness to engage in clinical interventions in Qatar. *Qatar Med J.* (2021) 2020:47. 10.5339/qmj.2020.47 33598417 PMC7863707

[21] Valdes A Walter J Segal E Spector T. Role of the gut microbiota in nutrition and health. *BMJ.* (2018) 361:36–44. 10.1136/bmj.k2179 PMC6000740 29899036

[22] Albury C Pope C Shaw S Greenhalgh T Ziebland S Martin S Gender in the consolidated criteria for reporting qualitative research (COREQ) checklist. *Int J Qual Heal Care.* (2021) 33:18–9. 10.1093/intqhc/mzab123 34428303

[23] Neubauer B Witkop C Varpio L. How phenomenology can help us learn from the experiences of others. *Perspect Med Educ.* (2019) 8:90–7. 10.1007/s40037-019-0509-2 30953335 PMC6468135

[24] Moisey L Campbell A Whitmore C Jack S. Advancing qualitative health research approaches in applied nutrition research. *J Hum Nutr Diet.* (2022) 35:376–87. 10.1111/jhn.12989 34997658

[25] Braun V Clarke V. Using thematic analysis in psychology. *Qual Res Psychol.* (2006) 3:77–101. 10.1191/1478088706qp063oa 32100154

[26] Condon L Bedford H Ireland L Kerr S Mytton J Richardson Z Engaging gypsy, roma, and traveller communities in research: maximizing opportunities and overcoming challenges. *Qual Health Res.* (2019) 29:1324–33. 10.1177/1049732318813558 30600758 PMC7322935

[27] Buettner D Skemp S. Blue zones: lessons from the world’s longest lived. *Am J Lifestyle Med.* (2016) 10:318–21. 10.1177/1559827616637066 30202288 PMC6125071

[28] Leeuwendaal N Stanton C O’Toole P Beresford T. Fermented foods, health and the gut microbiome. *Nutrients.* (2022) 14:1527. 10.3390/nu14071527 35406140 PMC9003261

[29] Mohr A Gumpricht E Sears D Sweazea K. Recent advances and health implications of dietary fasting regimens on the gut microbiome. *Am J Physiol Gastrointest Liver Physiol.* (2021) 320:G847–63. 10.1152/ajpgi.00475.2020 33729005

[30] Baty V Mougin B Dekeuwer C Carret G. Gut health in the era of the human gut microbiota: from metaphor to biovalue. *Med Heal Care Philos.* (2014) 17:579–97. 10.1007/s11019-014-9552-2 24610296

[31] Barqawi H Adra S Ramzi H Abouaggour M Almehairi S. Evaluating the knowledge, attitudes and practices of the UAE community on microbiota composition and the main factors affecting it: a cross-sectional study. *BMJ Open.* (2021) 11:e047869. 10.1136/bmjopen-2020-047869 34404705 PMC8372808

[32] Kumar H Salminen S Verhagen H Rowland I Heimbach J Bañares S Novel probiotics and prebiotics: road to the market. *Curr Opin Biotechnol.* (2015) 32:99–103. 10.1016/j.copbio.2014.11.021 25499742

[33] Khalesi S Bellissimo N Vandelanotte C Williams S Stanley D Irwin C. A review of probiotic supplementation in healthy adults: helpful or hype? *Eur J Clin Nutr.* (2019) 73:24–37. 10.1038/s41430-018-0135-9 29581563

[34] Khalesi S Vandelanotte C Thwaite T Russell A Dawson D Williams S. Awareness and attitudes of gut health, probiotics and prebiotics in australian adults. *J Diet Suppl.* (2021) 18:418–32. 10.1080/19390211.2020.1783420 32588677

[35] Barnes K Ball L Desbrow B Alsharairi N Ahmed F. Consumption and reasons for use of dietary supplements in an Australian university population. *Nutrition.* (2016) 32:524–30. 10.1016/j.nut.2015.10.022 26819063

[36] Singh R Chang H Yan D Lee K Ucmak D Wong K Influence of diet on the gut microbiome and implications for human health. *J Transl Med.* (2017) 15:73. 28388917 10.1186/s12967-017-1175-y PMC5385025

[37] Dinu M Pagliai G Casini A Sofi F. Mediterranean diet and multiple health outcomes: An umbrella review of meta-analyses of observational studies and randomised trials. *Eur J Clin Nutr.* (2018) 72:30–43. 10.1038/ejcn.2017.58 28488692

[38] Medina F. Looking for commensality: On culture, health, heritage, and the mediterranean diet. *Int J Environ Res Public Health.* (2021) 18:2605. 10.3390/ijerph18052605 33807765 PMC7967324

[39] Díez J Bilal U Franco M. Unique features of the Mediterranean food environment: Implications for the prevention of chronic diseases Rh: Mediterranean food environments. *Eur J Clin Nutr.* (2019) 72:71–5. 10.1038/s41430-018-0311-y 30487563

