# Supplementary material for: Older adults and healthcare professionals have limited awareness of the link between the Mediterranean diet and the gut microbiome for healthy aging
Source: Front Nutr. 2023 Jan 27;10:1104238. doi: 10.3389/fnut.2023.1104238 (PMC9911522; doi:10.3389/fnut.2023.1104238)
Supplement: Supplementary file 1 [file Data_Sheet_1.docx]

Supplementary Material

**Supplementary File 1. Topic Guide for Older Adults**

Please note: This is an indicative list of questions which may not be entirely pursued in the order below. Follow-up questions to seek further understanding of particular issues may also be asked.

**Health and Diet**

- Can people’s lifestyles influence their future health? And if so, what are the best ways to stay healthy? Prompt: Exercise, diet, smoking, crosswords, etc? to achieve healthy ageing, in your opinion how important is diet relative to exercise, smoking, alcohol intake, etc?
- *Additional Q For organised groups only: What influences the focus on a healthy diet in your organisation/group?*
- Are you familiar with the Irish Healthy Eating Guidelines; or ‘the food pyramid’? What do you consider is a “healthy diet”? Do you follow this diet, or any other particular diet? Why/why not?
- What makes it easier or harder to follow a “healthy diet”? What could help to overcome these challenges?
- Have you heard of the Mediterranean diet? (Explanation if not familiar: lots of fresh fruit and veg; oily fish but low red meat; includes olive oil). What do you think of this type of diet relative to other diets in terms of the potential health benefits? And easiness/hardness to follow?

**Gut Microbiota**

- Have you heard of a healthy gut? And gut microbiota? What do these terms mean to you? If familiar: (Explanation if not familiar: A’ healthy gut’ contains healthy bacteria and immune cells that ward off infections and send signals to the body and brain which help maintain general health. ‘Gut microbiota’ are the microorganisms that live in our gut- like bacteria and fungi). Having heard this, in your opinion, how important is maintaining healthy gut microbiota?

**Factors Influencing Health and Diet**

- How do you decide what to purchase at the supermarket? And when deciding what to eat at home? Prompt if needed: habit, convenience, health benefits, cost, other
- Who else influences what you eat and how? (eg family do the shopping/cooking; home help/ meals on wheels)?
- Where do middle aged and older people get their health information from (prompt: internet; newspapers; radio; TV; GP; other HCPs; family; peers, other). Prompt: is this the same for diet information as for other health information?

**Food Products for Health**

Explain: A ‘food product’ is a processed food made from several ingredients, like a sliced pan, or a lasagne or a yoghurt, not an egg or a bag of apples that is just gathered and packaged. Some food products in the supermarket are marketed as having a health benefit. (Explain if needed that not talking here about prescribed nutritional drinks picked up from a pharmacy, but about food products available in supermarkets).

- What do you think of these food products? (Give specific examples if needed: e.g. cholesterol lowering drink or margarine, probiotic yoghurts, protein bars, etc). In your opinion, where do these food products fit into an overall healthy diet? What would prompt you to buy a food product with health benefits?
- For you, what should be the key necessary features of a food product that is aiming to improve health? (Prompt: cost, taste, availability locally, evidence for benefit, personal relevance to them, convenience, packaging size, shelf-life, recyclable packaging?) Which is most important within these?
- Thinking of a food product that you might buy hoping to improve your health, would you prefer it to be a drink or a food? If a drink, would you prefer sweet or savoury? And if a food? For food: is texture important (eg would you prefer a firm bar or soft muffin or something crunchy, etc, or is this important if you think it is good for you)?
- Is important to you that the protein in a food product would come from a dairy source (so milk-based) or from a plant base (like soy or chickpea)? Why do you say this?
- What else should food product developers be considering for middle aged and older people?
