# Supplementary material for: Older adults and healthcare professionals have limited awareness of the link between the Mediterranean diet and the gut microbiome for healthy aging
Source: Front Nutr. 2023 Jan 27;10:1104238. doi: 10.3389/fnut.2023.1104238 (PMC9911522; doi:10.3389/fnut.2023.1104238)
Supplement: Supplementary file 2 [file Data_Sheet_2.DOCX]

Supplementary Material

# Supplementary File 2. Topic Guide for Healthcare Professionals (HCPs)

Please note: This is an indicative list of questions which may not be entirely pursued in the order below. Follow-up questions to seek further understanding of particular issues may also be asked.

**Health and Diet**

- What lifestyle factors influence peoples’ health in later life? (Prompt: Exercise, diet, smoking, doing crosswords, etc)
- Within these, how important is diet relative to other factors for late middle-aged and older people who want to improve their health?
- What do you consider is a “healthy diet” for late middle aged and older people to prevent frailty, dementia, inflammation etc? What are the most important components? Do these differ according to the health issue in focus, and if so, how? Are you familiar with the national Healthy Eating Guidelines? And the Food Pyramid? What do you think of these? Do you think middle aged and older people follow them?
- What do you think of the Mediterranean diet versus a “healthy diet” ? (Explanation if not familiar: lots of fresh fruit and veg; oily fish but low red meat; includes olive oil). What is different? What is better or missing from it?
- How important is the gut microbiome in overall health? (Explanation if not familiar: ‘Gut microbiota’ are the microorganisms that live in our gut- like bacteria and fungi).

**Factors Influencing Health and Diet**

- What influences older people’s dietary choices? Prompt if needed: habit, convenience, health benefits, cost, other. *Can consider separately ‘at supermarket’ and ‘when deciding what to have for a meal at home’.*
- Do they make these food choices personally, or are others also influential, and how?
- What are the facilitators and challenges for middle aged and older people in following a “healthy diet”? What is different for the Mediterranean diet versus other healthy diets?
- What could help to overcome these challenges? 🡪 eventually prompt re. food products

**Food Products for Health**

- What is your attitude to food products in supermarkets that are marketed as having a health benefit? Where do they fit in in overall diet and overall lifestyle? What prompts middle aged and older people to buy a food product marketed as having health benefits?
- (A ‘food product’ is a processed food made from several ingredients, like a sliced pan, or a lasagne or a yoghurt, not an egg or a bag of apples that is just gathered and packaged).
- What are the key necessary features of a food product that is aiming to improve health for it to be attractive to consumers? Prompt: cost, taste, evidence for benefit health benefit, personal relevance to consumer, convenience, pack size, shelf-life, recyclable packaging? Which is most important within these?
- Why are most prescribed oral nutritional products for older people drink based and sweet?
- Is important that the protein in a food product is dairy (e.g. milk) or plant based (e.g. soy, chickpea)?
- What else should food product developers be considering specifically for older people to improve the appeal of the product? And the benefit of the product?
