# Supplementary material for: Older adults and healthcare professionals have limited awareness of the link between the Mediterranean diet and the gut microbiome for healthy aging
Source: Front Nutr. 2023 Jan 27;10:1104238. doi: 10.3389/fnut.2023.1104238 (PMC9911522; doi:10.3389/fnut.2023.1104238)
Supplement: Supplementary file 3 [file Data_Sheet_3.docx]

Supplementary Material

# Supplementary File 3. Themes, sub-themes and illustrative quotations

| **Domain** | Theme | *Illustrative Quotation* |
| --- | --- | --- |
| **Perception of the Mediterranean Diet** | Foods considered to form part of the MD | - *“The Mediterranean diet sounds great, but I wouldn't think it includes all the red wine and sugars … even pastas … a clear definition of the MD is hard to get … is the MD then greens? Is it just vegetables? … and lots of oil?” (OA#30).* - *“it’s' based on multi coloured vegetables, healthy oils like your omega 3 oils, oily fish, limited amounts of meat” (OA#21)* - *“I still think we need a little bit of red meat to sustain us” (OA#49).* - “*I suppose they eat a lot of fish, and they grow their own food. I don't know necessarily what the food is, I think there's nuts involved?” (OA#4)*. - “*When we were growing up our mother used to spoon us cod liver oil … (it’s) good for your bones and builds you up”* *(OA#55)* - *“Over the years I've heard of people taking spoonfulls of virgin olive oil or something like that to stave off dementia” (OA#44).* - *“I always think oil is unhealthy, even olive oil … that’s just oil? you're eating lots of fats?” (OA#4)* - *“…in the Mediterranean diet, it's ok to eat cheese, use cream, butter … there's no problem with them.” (OA#16).* |
|  | Perceived benefits of the Mediterranean Diet to health | - *“I think there’s some place in Italy where they all live to a hundred or something and it’s supposed to be something to do with their diet they reckon.” (OA#20)* - *“I think that it has beneficial effects on, let’s say if you have high cholesterol.” (OA#28)* - *“the general healthy eating with the Mediterranean slant on it seems to be the most … that there's most information there with regards to prevention of dementia” (Dietitian #4)* - *“as patients are moving to moderate and advanced stages of dementia, it's whatever you can get into them and focusing on high calorie and high protein” (Geriatrician #4)* - *“… certain lifestyles or behaviours that are not related specifically to the type of food that you eat … but can be around how you organise mealtimes” (Geriatrician #6)* - *“I would have thought the evidence starts with these populations where it is their whole-food diet that they've had that's helped to prevent against whatever diseases” (Dietitian #5)* - *“If you extract the social component of the MD and just give people the Mediterranean foods…is it still beneficial?” (Pharmacist #15)* - *“The problem with diet and relating it to chronic illness prevention, the studies are far few and between and not robust in some cases” (Dietitian #4)* - *“from a consumer's point of view, I never saw any evidence that it (MD) will extend my life” (OA#30).* |
|  | | |
| **Perception of the gut microbiota** | The gut microbiota is a new and emerging science | - *“I know that there's a lot of research going on now on the gut microbiome whereas that wouldn't have been happening at all in my day. Or maybe it was starting to happen? But I wasn't at all aware of it. Whereas I am now … and I would wonder about certain things, would they be helpful for me … like the probiotics” (OA#10)* - *“I've heard a lot about it on the radio, I have heard dietitians and nutritionists and people talking about how important it is to have a balanced microbiome is it?” (OA#5)* - “*we are aware that there in UCC* (University College Cork) *… doing terrific work on the gut … and relating it to the brain and depression*” *(OA#28).* - *“There’s so much evidence coming out now and I don’t know about you, but I’ve been struggling to keep on top of it” (Dietician #3)* - *“You see a lot of people with irritable bowel syndrome … and that’s related to anxiety and the gut… I think having that research about the gut microbiome is really important for all round health, not just for digestive health, but mental health” (Dietitian #3).* |
|  | Perceived health benefits are “not common knowledge” | - *“there's just so many things that it's (gut microbiota) affected and that's not common knowledge” (Dietitian #1)* - *“When you say second brain, I don't quite understand the relationship between healthy gut and healthy body, and not ageing and dementia … how far is it actual proved scientific knowledge … that the healthy gut does prevent dementia? Or is it a theory that you're still working on?” (OA#63)* - *“there’s certain foods which … if I eat them, I feel it puts me in a bad mood” (OA#4)* - *“I have read about it … there’s some bacteria in the brain … but I let the details go” (OA#64)* - *“I wouldn't have made the connection with dementia.”* *(OA#52)* - *“it would be an absence of indigestion and digestive disorders.” (OA#61)* - “*you would be looking at fibre and fluid intake, not necessarily very probiotic effective food, but just the overall fibre their diet provides” (Dietitian #7)* - *“Anything I see in the line of live cultures … I usually try … those yoghurts and things now with live cultures. I don't know what it actually means but I presume it's something fairly good.” (OA#46)*. - “*you go 12 hours to 14 hours with no food, isn't that another way of doing that … clearing your gut?” (OA#42).* - *“what [linseed] does is it releases the oil into the gut I think and it helps to take out some of the food, the sort of … the poisons … the bad stuff” (OA#31).* - “*there’s so many things that it affects … but they’re (older adults) not aware of the other general health benefits to everything, that it's not just gut*” (*Dietician#1)* - “*there's some good bacteria and bad bacteria and if the good bacteria win, you won't have leaky gut and you won’t have inflammation” (OA#2)* - *“it certainly would be something I would think is important in an older well cohort, where you're looking at their overall health, not just their malnutrition risk and management” (Dietician #4)* |
|  | Probiotic supplements considered for use ‘every now and then’ | - *“I look after my biome by … taking a supplement and eating live yoghurt” (OA#21)* - *“I would have thought … the antibiotics have done so much damage to your system and then the probiotics, you take them to counteract that.” (OA#1)* - *“I kind of assume at this stage that my gut is probably a bit leaky, I think as we get older … so every so often I'll take a product that is meant to kind of help repair the gut a bit.” (OA#25)* - *“Am I going to start changing the way I eat to try and tweak my gut microflora? probably not … I don’t think I’ve got a problem, so I don’t think I need to do anything about it” (OA#3)* - *“when you take some of the probiotics, many of them, well anecdotally anyway, are dead by the time they reach your small intestine … where they're needed. So maybe it's that I'm biased because, again ultimately, I often feel that it's about using people's vulnerabilities to make money. And that's what I'm cynical about” (Geriatrician #5).* |
|  | | |
| Challenges in promoting a Mediterranean diet for gut microbiota benefit for healthy ageing | Little awareness of the Mediterranean diet and microbiota linkage for healthy ageing | - *“Most fruits I'm sure are good because they keep the tummy active” (OA#36)* - “*fibre* *is what feeds the microbiome” (OA#16)* - *“… I don’t think I’d associate yoghurts now with Mediterranean diet but apparently, they’re supposed to be good for your gut as well.” (OA#20)* - *“I have my red cabbage sauerkraut because of the bacteria, the good bacteria … the bionics, or biotics” (OA#64)* - *“The ultimate healthy diet, I think you'd be looking at Japanese macrobiotic sort of diet … Whereas the Mediterranean diet, well it's tasty … and a glass of wine with it …. I just think it's (MD) a nice way to enjoy food, as opposed to we'll say the next level of healthiness” (OA#19)* |
|  | Acceptability: Socio-cultural and habitual differences of the Mediterranean Diet; ‘it's not what they're used to’. | - *“It's something different from what we'd be used to*” *(physiotherapist #10)* - *“I'm not sure that many Irish people would be able to cope with eating as late in the evening as they do” (OA#24)* - *“I'm not a great fan of oily fish … I suppose I'm more a meat and two veg sort of fellow” (OA#21)* - *“it's the fish on Friday. But will you have fish on Tuesday? You might need a little bit of encouragement” (Meal-delivery service coordinator #17)* - *“… potato salad and coleslaw … all the mayonnaise … It's disgusting. I think in Ireland a lot of people see that as a salad” (OA#1)* - “*no salads in the Winter, no cold food … your gut doesn’t like it in the winter” (OA#23)* - *“what's wrong with having an Irish diet and looking at our own seasonal foods and maybe bringing something that doesn't have to travel as far. I mean we have lovely cabbage and Brussels sprouts” (OA#26).* |
|  | Accessibility: The Mediterranean Diet in different food environments | - *“The fruit and veg, seasonality …. if you're in the Mediterranean, there would be more opportunities to eat fresh, it's more accessible” (Dietitian#4)* - *“The affordability of a Mediterranean diet and the effort required to accumulate the parts of it might be a barrier for people on reduced income and with people with reduced mobility. Maybe not everything is readily available in their local corner store” (Pharmacist #15).* |
|  | Tailoring communication to promote the Mediterranean diet and gut microbiota | - *“if I went on the Mediterranean diet…” (OA#3)* - *“on the Mediterranean diet, does it have to be full Mediterranean diet or can it be incorporated?” (OA#S20)* - *“It's overlapping with our, say, increase fish, get your fruit and veg in there. It's indirectly Mediterranean without calling it that” (Dietitian #1).* - *“If you go diving into olive oil, chickpeas, and lentils … it just won’t work for the person. It’s just too much too fast and too many words” (Dietitian #3)* - *“… it’s not necessarily something I mention to them, but it's in my head when I'm talking about getting the balance right … if they were talking about the good stuff in the yoghurt … I would talk about the probiotic, again not the technical terms” (Dietitian #1)* |
